# Does combining estradiol cypionate and GnRH for ovulation induction in recipient cows increase pregnancy rate after timed embryo transfer?

**DOI:** 10.1590/1984-3143-AR2022-0067

**Published:** 2022-10-17

**Authors:** Wagner Marques Lima, Fabiane Pereira de Moraes, Rogério Ferreira, Rafael Gianella Mondadori, Arnaldo Diniz Vieira, Nathália Wacholz Knabah, Danylo Cintra Medeiros Lima, Monique Tomazele Rovani, Luiz Francisco Machado Pfeifer, Paulo Bayard Dias Gonçalves, Bernardo Garziera Gasperin

**Affiliations:** 1 Programa de Pós-graduação em Veterinária, Faculdade de Veterinária, Universidade Federal de Pelotas, Capão do Leão, RS, Brasil; 2 Biotec Serviços de Apoio à Pecuária, Protásio Alves, RS, Brasil; 3 Faculdade de Zootecnia, Universidade do Estado de Santa Catarina, Chapecó, SC, Brasil; 4 Universidade Federal do Rio Grande do Sul, Porto Alegre, RS, Brasil; 5 Embrapa Rondônia, Porto Velho, RO, Brasil; 6 Universidade Federal do Pampa, Uruguaiana, RS, Brasil

**Keywords:** gestation, luteal function, progesterone, synchronization

## Abstract

Estradiol cypionate (EC) or GnRH have been widely used for ovulation induction in timed embryo transfer (TET). EC administration increases the proportion of cows that show estrus, whereas GnRH promotes more synchronized ovulations. The aim of the present study was to evaluate the potential beneficial effects of combining EC and GnRH in TET. In experiment 1, no difference was observed on serum progesterone concentrations on Day 6 and 13 after GnRH treatment between GnRH and EC+GnRH groups. In experiment 2, pregnancy per embryo transfer (P/ET) did not differ (*p* = 0.69) between GnRH (62.8%) and EC+GnRH (58.7%) groups. In conclusion, combining EC and GnRH for ovulation induction does not increase progesterone secretion and pregnancy rate after TET in cattle.

## Introduction

In Brazil, there are several alternatives for ovulation induction in cows, including estradiol benzoate (EB), estradiol cypionate (EC) and GnRH. Although EC has been widely used at progesterone device withdrawal, eliminating the need for animal handling before timed artificial insemination (TAI), there is greater variation in the time of ovulation compared to GnRH treatment (Souza et al., 2009). In contrast, administering estradiol at proestrus regulates endometrial gene expression (Sá et al., 2017) and increases the proportion of cows that show estrus (Pfeifer et al., 2020), which are more likely to become pregnant after TAI (Barbosa et al., 2022) or TET (Frade et al., 2014).

Previous studies have compared different treatments for ovulation induction in cows submitted to TAI, as reviewed by Consentini et al. (2021). However, to our knowledge, there is no study evaluating the effect of combining the potential beneficial effects of EC and GnRH in timed embryo transfer (TET). The aim of the present study was to evaluate the effect of combining EC and GnRH for ovulation induction on luteal function and pregnancy rate after TET in cows.

## Methods

All procedures involving animals were approved by the Ethics Committee on Animal Experimentation of the Federal University of Pelotas (CEEA-UFPel #57360).

### Experiment 1

Non-pregnant, non-lactating, Jersey and Holstein cows (n=12) were submitted to a hormonal protocol, based on the insertion of a progesterone (P4) intravaginal device (IVD) (1 g, Primer, Agener União) and an intramuscular (i.m.) injection of 2 mg EB (Agener União) on Day – 11 (D-11). On D - 4, i.m. injections of 482 µg of cloprostenol sodium (Estron, Agener União) and 300 IU eCG (SincroeCG; Ourofino) were administered. On D -2, the IVDs were removed, and half of the cows (n=6) received an i.m. injection of 0.6 mg EC (ECP, Zoetis). On Day 0, all the cows received an i.m. injection of GnRH analog (10 µg buserelin acetate; Sincroforte, Ourofino), and the ovary of the pre-ovulatory follicle was determined by ultrasound. On Days 6 and 13 after GnRH, the animals were submitted to ultrasound to verify the presence of a corpus luteum (CL), where the pre-ovulatory follicle was previously located, and blood samples were collected for progesterone assay. Progesterone data were analyzed by paired Student’s T test, using cow as subject.

### Experiment 2

This experiment was performed in two replicates. Non-pregnant, suckling crossbred Angus cows (n=184), between 35 and 80 days postpartum and with body condition score (BCS) 3 to 4 (scale 1 to 5) were submitted to a hormonal protocol. On Day -10, the cows received an IVD containing 1 g of P4 (Reproneo, GlobalGen) and an i.m. injection of 2 mg EB (Bioestrogen; Biogénesis Bagó). On Day -2, 150 µg of d-cloprostenol (Croniben, Biogénesis Bagó), 400 IU of eCG (Ecegon; Biogénesis Bagó) were administered i.m. and the IVDs were removed, and half of the cows (n=92) received an i.m. injection of 0.6 mg EC (Cipiotec, Agener União). On Day 0, all the cows received an i.m. injection of GnRH analog (10 µg buserelin acetate - Gonaxal, Biogénisis Bagó). On Day 7, cows with a CL and without reproductive disorders received a single fresh or thawed *in vivo* produced embryo (grade 1 or 2; morula or initial blastocyst) in the uterine horn ipsilateral to the CL. The embryo transfers were conducted in a commercial farm, and the embryos were collected from 21 donor cows, which were mated with 11 bulls, selected by the owner. Fifty-six days after embryo transfer, pregnancy diagnosis was performed through transrectal ultrasonography. Pregnancy data were analyzed by logistic regression including group, replicate and embryo type (fresh or thawed) as fixed effects.

## Results

### Experiment 1

**Figure 1:**
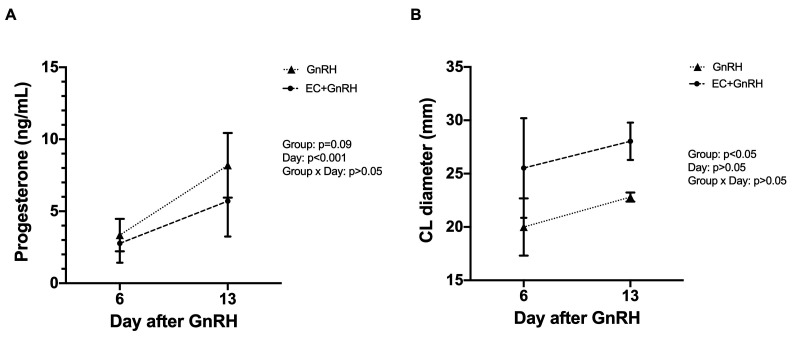
(A) Serum progesterone concentration and (B) Corpus Luteum (CL) diameter in cows treated with GnRH or EC+GnRH for ovulation induction. Estradiol cypionate (EC) was administered at intravaginal device withdrawal in cows from EC+GnRH group, whereas GnRH treatment was performed 48 h later in both groups.

Two cows from each group did not ovulate and were excluded from the trial. There was no significant difference (*p* = 0.09) between GnRH and EC+GnRH groups in progesterone concentration on Days 6 and 13 after GnRH treatment (Figure 1). Cows from EC+GnRH had larger CL diameter (*p* < 0.05) compared to GnRH group (Figure 1).

### Experiment 2

**Figure 2:**
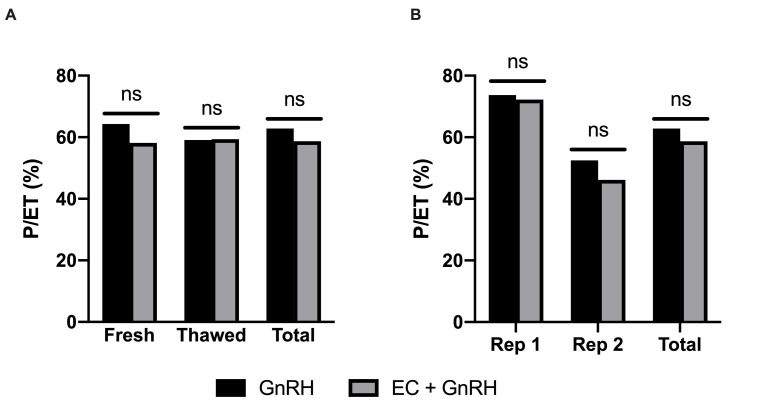
(A) Pregnancy rates according to embryo source (fresh or frozen thawed) and (B) replicate for GnRH and EC+GnRH groups. Estradiol cypionate (EC) was administered at intravaginal device withdrawal in cows from EC+GnRH group, whereas GnRH treatment was performed 48 h later in both groups. Rep = replicate; ns = p > 0.05.

The recipient utilization rates (83.1%) did not differ (*p* = 0.55) between GnRH (84.8%) and EC+GnRH (81.5%) (Table 1). Pregnancy per embryo transfer (P/ET) rate did not differ between groups when fresh or thawed embryos were transferred (*p* = 0.46; Figure 2). Although significant difference was observed in P/ET rate between replicates (*p* = 0.002), there was no difference between groups (*p* = 0.69; Figure 2).

**Table 1:** Recipient utilization rates and pregnancy rates according to each treatment.

**Farm**	**Group**	**TOTAL**	**ET**	**%ET**	**Pregnant**	**P/ET**	**P/TOTAL**
Farm 1	EC+GnRH	43	36	83.7	26	72.2	60.5
	GnRH	43	38	88.4	28	73.7	65.1
Farm 2	EC+GnRH	49	39	79.6	18	46.2	36.7
	GnRH	49	40	81.6	21	52.5	42.9
Overall	EC+GnRH	92	75	81.5	44	58.7	47.8
	GnRH	92	78	84.8	49	62.8	53.3

ET: embryo transfer; %ET: recipient utilization rate; P/ET: pregnancy per embryo transfer; P/TOTAL: total pregnancy rate.

## Discussion

Our results show that combining EC and GnRH for ovulation induction does not affect progesterone synthesis and pregnancy rate after TET. To our knowledge, this is the first study evaluating potential effects of combining EC and GnRH for synchronizing embryo recipients. The lack of difference on progesterone concentrations was expected because a previous study in dairy cows demonstrated that both EC and GnRH are efficient in inducing ovulation (Souza et al., 2009). Furthermore, both groups received GnRH treatment, which promotes more synchronized ovulations compared to EC alone (Barbosa et al., 2022).

Our results are in agreement with a previous study that compared EC, EC+GnRH or GnRH for ovulation induction in a progesterone-estradiol based protocol, which also did not observe differences in pregnancy rate on days 31 and 60 after TAI, reviewed by Consentini et al. (2021). It is well established that EC administration induces estrous behavior (Pfeifer et al., 2020) and that recipient cows that show estrus are more likely to become pregnant (Frade et al., 2014; Bó and Cedeño, 2018). Although estrus detection was not performed in the present study, treatment with EC had no beneficial effect on the percentage of cows that received an embryo and on pregnancy rate. The recipient utilization rates for both groups were above 80%, which are similar to the rates reported in other studies (Pérez-Mora et al., 2020). It is noteworthy that, regardless of the group, P/ET rates were close to 60%, which indicates the high quality of the *in vivo* produced embryos and the high fertility of the recipient cows used in the study (Hasler, 2014; Bó and Cedeño, 2018). Further studies are necessary to investigate potential effects of EC in cows under challenging circumstances, with low BCS, since these cows are less likely to show natural estrus (Pfeifer et al., 2021) and, consequently, more likely to benefit from EC treatment.

## Conclusion

Combining EC and GnRH for ovulation induction does not increase progesterone secretion and pregnancy rate after TET in cattle.
